# Review of the Imaging Modalities in the Gynecological Neoplasms During Pregnancy

**DOI:** 10.3390/cancers17050838

**Published:** 2025-02-28

**Authors:** Gabriele Masselli, Charis Bourgioti

**Affiliations:** 1 Radiology Department, Umberto I Hospital, Sapienza University, Via del Policlinico 155, 00161 Rome, Italy; 2 School of Medicine, National and Kapodistrian University of Athens, Aretaieon Hospital, 11527 Athens, Greece

**Keywords:** cancers, pregnancy, imaging, MRI, gynecology, cervix, ovaries, uterus

## Abstract

The aim of this review is to provide an update on the imaging triage, safety considerations, and cancer-imaging features of common and uncommon gynecological tumors during pregnancy. Clinical examination can be inconclusive, especially for the evaluation of intra-abdominal diseases since symptoms related to cancer such as fatigue, vomiting, or nausea overlap with common pregnancy-associated symptoms. Delayed pregnancy, advanced maternal age, and improvements in diagnostic imaging in developed countries have increased the incidence of cancer diagnosis in gravid populations. Non-invasive diagnostic imaging has a central role in the initial diagnosis and management of pregnancy tumors. Sonography is the first modality for the abdominal evaluation of gravid patients because it is widely available, low cost, and lacks adverse effects on the mother and fetus. MR imaging is considered the imaging modality of choice for diagnosis and staging of gynecologic cancer during pregnancy due to its larger imaging field of view, better reproducibility, and excellent soft tissue contrast. Moreover, whole-body diffusion-weighted imaging (DWI) has been proposed for the oncologic staging of pregnant patients; however, large prospective studies are needed to support its clinical implementation. Gynecological cancer diagnosed during pregnancy requires accurate diagnosis and staging to determine optimal treatment based on gestational age, and therefore multidisciplinary team collaboration is pivotal.

## Simple Summary

Cancer in pregnancy is a challenging issue due to its extreme emotional impact and concerns on mother and fetus health. The most frequent gynecological cancer diagnosed during pregnancy is cervical cancer, followed by ovarian, uterine, and vulvar tumors. Prompt diagnosis and accurate staging are mandatory in the management of these conditions and multidisciplinary care is important, involving both gynecologists and gynecological oncologists, along with radiologists and radiation oncologists, in order to choose the best treatment approach based on the imaging findings.

## 1. Introduction

Cancer in pregnancy is defined as any cancer that occurs during pregnancy or in the first postpartum year. It is the second leading cause of mortality in women of reproductive age [1,2]. Management of pregnancy-related cancer is a challenging issue due to its extreme emotional impact and concerns on mother and fetus health [3,4,5].

Delayed pregnancy, advanced maternal age, and improvements in diagnostic imaging in developed countries have possibly increased the incidence of cancer diagnosis during the last years [1,2,3,4,5,6,7].

Tumors in pregnancy are a rare occurrence, accounting for 0.02–0.1% of the total gravid population, and the incidence is even lower in developing countries due to younger mother age [8].

The rare and challenging case of cancer in pregnancy is becoming relatively more common in the developed world given the fact that the incidence of many malignancies begins to rise during the fourth decade of life [9].

The most frequent cancer diagnosed during pregnancy is breast cancer, followed by hematological malignancies and gynecologic tumors including cervical cancer, followed by ovarian, uterine, and vulvar tumors [10].

Prompt diagnosis is mandatory in the management of these conditions and multidisciplinary care is important, involving both gynecologists and gynecological oncologists, along with radiation oncologists, in order to choose the best treatment approach. Although termination of pregnancy does not improve prognosis, it may be considered if particular conditions are present [11,12]; thus, careful parent consultation and psychological support have an important role too.

Clinical examination can be inconclusive, especially for the evaluation of intra-abdominal disease since common symptoms related to cancer such as fatigue, vomiting, or nausea overlap with common pregnancy-associated symptoms [1,2,3,4,5,6,7].

Minimal interventional procedures such as endoscopy, bone marrow aspiration, lumbar puncture, or even laparoscopy and biopsies in specific sites (e.g., cervix), which may be required for cancer diagnosis, should be used with caution during pregnancy in order to avoid the risk of miscarriage [1,2,3,4,5,6,7].

Thus, non-invasive diagnostic imaging has a central role in the initial diagnosis and management of pregnancy tumors.

A literature search was conducted in the Medline database for articles in the English language published between 1991 and 2024 using the following keywords: cancers; pregnancy; imaging; MRI, US, gynecology; cervix; ovaries; uterus. Prospective or retrospective research studies and review articles were analyzed; the selection of the studies was based on the presence of the following criteria: appropriate study design, adequate study population, the use of clear diagnostic evidence and reliable statistical data, and reproducibility of the results; 106 studies were finally included in the present review report.

The aim of this review is to discuss the overall role of imaging and illustrate imaging features of common and uncommon gynecological tumors during pregnancy.

Key questions for this review were as follows: to discuss the advantages and drawbacks of various imaging modalities in evaluating gynecological neoplasms during pregnancy; to review the epidemiology and clinical management of gynecological neoplasms during pregnancy; and to focus on the imaging aspects of the most common gynecological cancers encountered in pregnant patients.

## 2. Imaging the Oncologic Pregnant Patient—General Principles

Pregnancy status alone does not seem to adversely affect maternal oncologic outcomes, and overall prognosis is not different from that in non-pregnant patients [9,10,11].

Therefore, when planning the diagnostic and staging work-up of pregnant patients with cancer, several parameters need to be addressed including fetal (closely related to gestational age) and maternal survival (closely related to tumor staging at the time of diagnosis and possibility of metastatic disease), as well as the desire of the mother to preserve the pregnancy and future fertility [12,13].

Theoretically, all currently available imaging modalities may be used for the evaluation of the pregnant patient; however, in practice, the modalities involving ionizing radiation should be carefully applied with respect to the safety of the fetus [14].

Usually, the absorbed doses for diagnostic imaging even for abdominal computed tomography (CT) are less than 50 mGy and, therefore, can be considered safe for the fetus. However, since there are potential risks of teratogenesis if the dose exceeds the cut-off of 50 mGy (or 100 mGy for other researchers), especially in the first two trimesters, such as issues related to the cumulative radiation and potential stochastic effects (i.e., the risk of carcinogenesis), the use of ionizing radiation is best avoided during pregnancy [14,15,16,17,18].

CT in gravid patients can be considered as an option only when the information required cannot be obtained by other nonionizing imaging modalities and only after careful risk–benefit assessment, always keeping the radiation dose to the fetus as low as reasonably achievable (ALARA principle) [14]. Appropriate consultation for possible side effects to fetal health and written informed consent from the patient are strongly advised.

Generally, 18F-fluorodeoxyglucose (FDG) PET/CT is a helpful imaging modality for staging and restaging oncologic patients. Its use is discouraged in pregnant patients because the fetus is exposed to both ionizing radiation and potential toxicity of the radiopharmaceutical, although the reported total absorbed fetal doses are less than the threshold of 50 mGy [19]. However, it may be performed after a careful risk–benefit assessment if the provided information is medically required; in such cases, according to Wisely, a joint initiative of the American College of Radiology, Radiological Society of North America, American Society of Radiological Technologists, and the American Association of Physicists in Medicine, an FDG dose reduction to 5 mCi is recommended to minimize fetal exposure [18] (Table 1).

PET/MR imaging may be an alternative option providing the metabolic information without the CT-related radiation, but its availability is still limited [15].

Sonography is the preferred imaging for the initial evaluation of the abdomen in a gravid patient, due to its wide availability, low cost, and lack of adverse effects to mother and fetus. Color Doppler imaging during the first trimester should be used prudently, since there is a theoretical risk for tissue heating; however, the use of appropriate settings for obstetric imaging minimizes such risk.

As gestation progresses, the enlarged gravid uterus obscures the pelvic organs and retroperitoneum, rendering sonography less suitable for examination of the abdomen in patients with gynecologic malignancies.

MRI is a reliable alternative for the evaluation of abdominal pathology in the pregnant population, and as it does not utilize ionizing radiation, it may be safely applied to these patients [20,21].

Some controversies have been raised due to radiopulse stimulation in high magnetic fields, and subsequent tissue heating, which may induce cell damage, especially in early pregnancy; however, the use of a specific absorption rate (SAR) less than W/Kgr, virtually minimizes this risk [20]. In addition, there are some theoretical risks of acoustic damage, due to the noise produced from gradient echo sequences, especially after the gestational week 24, but nothing has been documented for both 1.5 T and 3.0 T MRI fields [21]. The use of intravenous gadolinium (Gd) on MRI is discouraged as the Gd chelate crosses the placenta and enters the fetal circulation with virtually unknown effects on the developing fetus [21]. It also has been reported that the use of gadolinium contrast increases the risk of the exposed fetus to any rheumatologically, inflammatory, or infiltrative skin condition during childhood (adjusted HR 1.36, 95% CI 1.09 to 1.69) as well as stillbirth and neonatal death (adjusted RR 3.70, 95% CI 1.55 to 8.85) [21]. Therefore, the use of gadolinium should be limited to scenarios where maternal benefits outweigh the potential fetal risk [21].

An example of an MR imaging protocol used to evaluate gynecologic malignancies in pregnant patients is presented in Table 2.

Whole-body diffusion-weighted imaging (DWI) has been evaluated for the oncologic staging of pregnant patients, showing good results and eliminating the need for intravenous contrast; however, technical difficulties such as long scanning times, which may be associated with maternal discomfort, and a lack of large prospective studies currently limit its clinical implementation [22].

## 3. Imaging of Specific Gynecologic Pregnancy-Related Tumors

### 3.1. Cervical Cancer

#### 3.1.1. Epidemiology, Pathological Anatomy, and Clinical Management

Cervical cancer is the most common gynecologic malignancy in pregnancy with an estimated incidence of 0.8 to 1.5 cases per 10,000 births [1,2,3,4,5,6,7,23,24,25]. Only 1–3% of women diagnosed with cervical cancer are pregnant or postpartum at the time of diagnosis [25]. About one-half of these cases are diagnosed prenatally, and the other half are diagnosed within 1 year after delivery [26].

Recommendations include the routine performance of a Pap smear at the first prenatal visit; therefore, most (up to 70%) pregnancy cervical cancers are detected at an early stage with small-volume disease [2,27]. In the case of high-grade epithelial lesions, colposcopy and cervical biopsy may follow [27,28]. Cervical biopsy can be performed safely in any pregnancy trimester and will not increase the incidence of complications during pregnancy, abortion rate, or premature delivery rate; however, curettage is not recommended because it increases the abortion rate and premature delivery rate [29,30,31,32,33,34]. If the *biopsy is positive for cervical cancer, treatment options should consider the cancer stage*, gestational age, and patient’s desire to preserve pregnancy [34].

In all cases of biopsy-confirmed invasive cervical cancer, staging work-up is indicated.

Radical hysterectomy and termination of pregnancy with fetus in utero or cesarean radical hysterectomy are recommended by the International Federation of Gynecology and Obstetrics (FIGO) for stage IB1 cervical cancer in pregnancy [7]. Occasionally, when the expectant mother is willing to keep the pregnancy, the option of radical trachelectomy via vaginal or abdominal approach for cervical cancer stage up to IB1 under strict eligibility criteria (i.e., tumor volume < 2 cm, distance from internal cervical os > 1 cm, and lack of abnormal lymph nodes) may be considered [35,36].

Since pregnancy does not have a negative effect on cervical cancer prognosis, careful clinical and radiological follow-up is another treatment option for stage IB1 cervical cancer in pregnancy [36,37].

The evaluation of lymph node status is critical to the prognosis and management of cervical cancer, particularly in the setting of pregnancy. 

While lymphadenectomy with histologic evaluation is the gold standard, nodal decidual changes during pregnancy can be misdiagnosed as pathologic [26,27,28,29,30,31,32,33,34,35,36,37,38].

#### 3.1.2. Imaging

No-contrast pelvic MRI with multiplanar T2 weighted and sagittal diffusion images is highly indicated for locoregional tumor staging [39].

Tumors in non-pregnant patients appear T2 hyperintense compared with the cervical stroma [39]. However, during pregnancy, cervical stroma edema is normally T2 hyperintense, and may obscure the underlying tumor; DWI improves the distinction between the tumor and adjacent tissue, since malignant cervical tumors show diffusion restrictions, thereby facilitating tumor size and extent evaluation [39].

**Figure 1:**
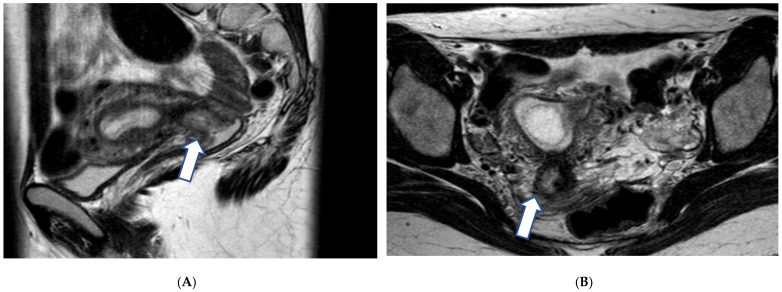
A 29-year-old gravid at gestational week 7, with biopsy confirming grade II squamous cell cervical cancer. T2-weighted images in the sagittal (**A**) and axial (**B**) plane demonstrate an ill-defined intermediate signal intensity mass within the cervical canal disrupting the low signal intensity stromal rim on the right (arrows).

MRI is essential for disease staging, as it can accurately assess important tumor characteristics including size (all three dimensions), vaginal, parametrial, or adjacent organ invasion in addition to hydronephrosis and lymph node involvement (Figure 1).

For early diseases limited to the cervix (International Federation of Obstetrics and Gynecology [FIGO] stage I), MRI helps with tumor delineation and provides a baseline for close follow-up and radical trachelectomy options [40].

For more advanced tumors (FIGO stage II or higher), MRI helps establish the local–regional extent of the adjacent tissues including parametrial fat, vaginal, pelvic, or bladder/rectal wall; the presence of which precludes surgical treatment.

In addition, radiologists should be aware of some MR imaging pitfalls. Post-biopsy hemorrhage may be a potential cause for false-positive DWI; to avoid this pitfall, it is recommended to wait at least 1 week before performing an MRI after cone-biopsy. Also, pelvic varices may mimic enlarged lymph nodes, so it is important to check DWI for the correct diagnosis [40].

Although transvaginal US has limited utility for the evaluation of cervical tumors since the cervix lies deep within the pelvis, in the hands of experts, it can provide information about tumor size and parametrial invasion when MRI is inaccessible [40].

Estimation of the distance between the tumor and the internal cervical os (ICO) is critical for planning uterine-sparing surgery; a tumor-to-ICO distance of more than 1 cm is usually required for successful surgical outcomes.

However, in pregnant patients, an ICO evaluation may be difficult since the gravid uterus distorts normal cervical anatomy and pregnancy-related edema can alter normal ICO T2 signal intensity; in such cases, the DWI feature is helpful for obtaining the correct evaluation.

Gadolinium contrast-enhanced imaging is usually performed in the postpartum period, as well as contrast-enhanced CT and PET/CT for staging.

New functional techniques including PET/MRI or WBDWI MRI can be useful examination tools for the presurgical evaluation of cervical cancer; however, large-scale studies are needed [15].

For patients with stage IB1 cancer or greater, low-dose chest CT may be considered to evaluate for pulmonary metastases. Imaging of the urinary tract for possible hydronephrosis by ultrasound or MRI is generally recommended [39,40].

MR imaging is the only examination that can reliably assess the response to treatment (surgical or chemotherapy) during pregnancy.

Some investigators propose an MR imaging examination every 4 weeks after diagnosis until the 30th week of pregnancy, to evaluate the course of disease and plan the cesarean section [39].

### 3.2. Ovarian Tumors

#### 3.2.1. Epidemiology, Pathological Anatomy, and Clinical Management

Incidentally, detected adnexal masses are common in pregnancy, discovered in approximately 2.8 to 11 of 100,000 pregnancies; however, the number of asymptomatic ovarian masses during pregnancy has been increasing because of the routine use of prenatal fetal sonography [2,41].

Most of these lesions are benign, with malignant tumors accounting for only 1% to 5% of the cases. Ovarian cancer is the second most common gynecologic cancer diagnosed during pregnancy, complicating 1 in 15,000 to 1 in 32,000 pregnancies [6,41,42,43,44,45,46].

In pregnant population germ cells, sex cord stromal and borderline histology are the most common malignancies [34], whereas epithelial ovarian cancer accounts for only 35% of the cases [36]. The majority of pregnancy-associated ovarian malignancies are diagnosed at an early stage, when the disease is still confined to the ovary [1,47].

Laboratory tests are not as useful compared to non-pregnant patients because tumor markers such as Ca-125, alpha-fetoprotein, HCG, and inibine can be normally elevated during pregnancy [36].

Identification of a benign adnexal mass is crucial since some of them discovered in the first trimester disappear spontaneously later on; therefore, an unnecessary surgical intervention may be avoided. However, the presence of an adnexal mass is the most common indication for gynecologic surgery during pregnancy, occurring in 0.1 to 2.4% of pregnant women [42,43,44].

Malignant germ cell tumors (MGCTs) are the most common type of ovarian malignancy diagnosed in pregnancy [44]. Dysgerminoma is the most common MGCT in pregnancy, comprising approximately 38%, followed by yolk sac tumors (30.4%) [45]. MGCTs are characteristically rapidly growing and unilateral; however, dysgerminoma may be bilateral in 10% of cases [1].

In the case of germ cells, a tumor unilateral adnexectomy with or without chemotherapy is usually performed [6,45,46,47,48,49].

In early-stage (I–II) invasive epithelial cancer, surgery with or without chemotherapy is usually performed, whereas in advanced-stage (III–IV) invasive epithelial cancers, the management depends on the gestational week: in patients with <20 gw, terminating pregnancy or neoadjuvant chemotherapy and cytoreduction can be considered, whereas, in patients with >20 gw, the preservation of pregnancy case by case is uncertain, and neoadjuvant chemotherapy and cytoreduction can be postponed until after delivery [38,50,51,52].

In other cases, such as in borderline tumors, conservative management postponing debulking surgery at the time of cesarean section is possible [53,54]. Surgical exploration is associated with an increased risk of miscarriage in the first trimester of pregnancy; hence, if possible, elective surgery should be deferred after 16 weeks of the second or third trimester, since the risk of abortion and preterm birth is lower [46,55,56,57,58,59,60,61,62,63,64,65,66].

#### 3.2.2. Imaging

The diagnostic accuracy of ultrasonography in discriminating between benign and malignant adnexal masses depends on the expertise of the sonographer [52,67]. Several imaging scoring systems have been developed to stratify the risk of malignancy and differentiate between benign and malignant adnexal tumors. The most popular system based on ultrasound features is the IOTA group Simple Rules [68,69] based on the presence or absence of five benign and five malignant ultrasound features. This system can correctly classify approximately 80% of the adnexal masses, with the rest of them being classified as indeterminate [69].

When the ovarian mass is large, the ultrasonography diagnosis is inconclusive, or there is an increased risk of malignancy, further imaging examination is needed.

**Figure 2:**
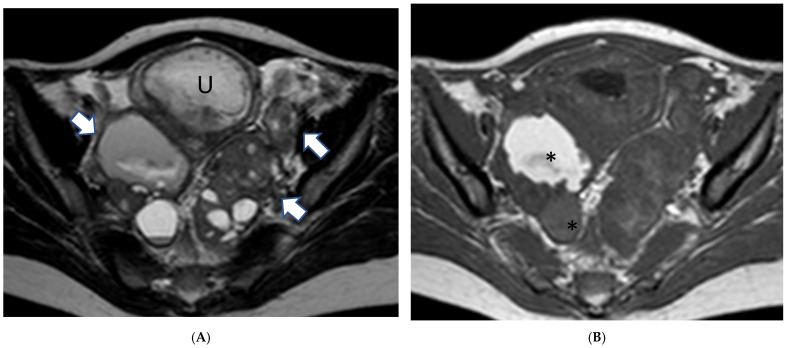
A 29-year-old woman at gestational week 9 presented with abdominal distention and fatigue: (**A**) Axial T2-weighted image demonstrates bilateral ovarian masses (arrows) with cystic and solid components. (**B**) Corresponding axial T1-weighted image shows high signal intensity content within some of the cystic compartments (*) indicative of hemorrhagic or high protein fluid. Low-grade serous cystadenocarcinoma of both ovaries was confirmed during surgery. U: gravid uterus.

Magnetic resonance imaging (MRI) is useful as an adjunct diagnostic tool when ultrasound is inconclusive [70,71,72] (Figure 2).

MRI might be useful for the evaluation of large masses that are difficult to visualize with ultrasound as it provides a larger field of view [73]. It can also be used for the presurgical evaluation of the extent of disease, peritoneal dissemination, and nodal metastases [73,74].

In addition, MR imaging is crucial for the diagnosis of acute complications of ovarian tumors including tumor bleeding, rupture, or torsion, and where emergency surgery is required [75]. The Ovarian-Adnexal Reporting and Data System (ORADS) MRI is a scoring system that has recently been proposed to assign the risk of malignancy of sonographically indeterminate adnexal masses in non-pregnant populations. ORADS includes six categories based on MRI features [76], and it is an accurate tool to predict malignancy in complex adnexal masses, with accuracies higher than 80% [76,77,78]. Currently, the ORADS MRI system cannot be applied to pregnant women since the use of gadolinium is strongly discouraged during pregnancy due to fetal safety issues. A recent study reported that non-contrast MRI proved a reliable tool to predict the risk of malignancy of adnexal masses in pregnant women and was extremely useful for inexperienced radiologists [79].

**Figure 3:**
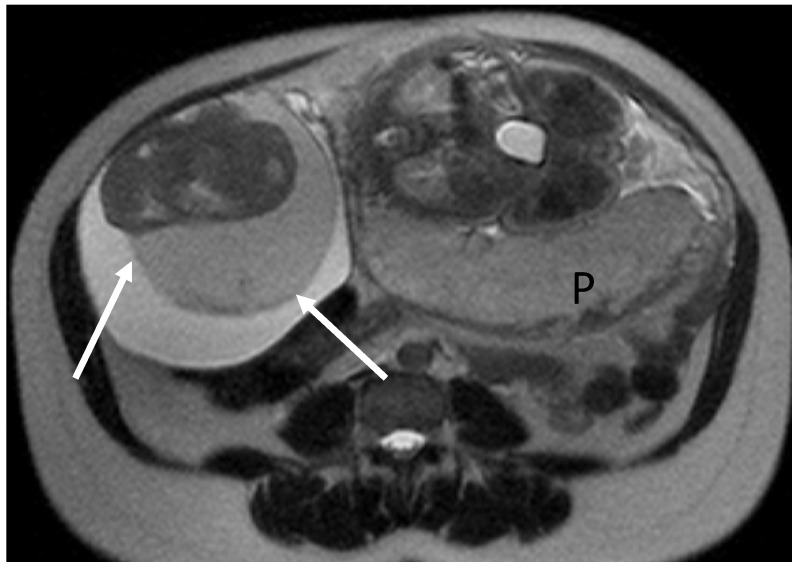
A 39-year-old pregnant woman at gestational week 30 presented with acute abdominal pain. Axial T2-weighted image demonstrates a large complex mass attached to the right uterine side (arrows). A mucinous cystadenocarcinoma of the right ovary was found during surgery. P: Placenta.

Thickened irregular walls or septa (i.e., diameter > 3 mm and highly vascular) with mural nodules and papillary projections, solid tissue, and necrosis are imaging findings indicative of borderline or malignant tumors while fatty tissue is generally associated with benign conditions (Figure 3) [80].

Large tumor size or the presence of abnormal lymph nodes, ascites, peritoneal deposit dissemination, and distant metastasis are also highly suspicious findings of malignancy [81].

#### 3.2.3. Mimickers of Malignancy

The differentiation between benign and malignant neoplasms is challenging because the latter may undergo pregnancy-related morphologic changes mimicking malignancy [52]. Typical examples of hormone-related mass during pregnancy include the corpus luteum of pregnancy, theca lutein cyst, and decidualized endometriomas [52,67,68,69,70].

Corpus luteum cysts result from the failure of involution of the corpus luteum, which produces progesterone during the first 8–9 weeks until replaced by the placenta [58].

Hyerreactio luteinalis (lutein cysts) is an uncommon condition due to increased levels of beta-hCG and appears as bilateral, multicystic ovarian masses, which can mimic ovarian hyperstimulation syndrome or mucinous borderline tumors. It is often associated with gestational trophoblastic disease and is only rarely found in normal uncomplicated pregnancies [82]. Theca lutein cysts generally resolve after gestational week 18, although a few may persist until after delivery [58].

Luteoma of pregnancy is a lesion consisting of proliferating luteinized stromal cells, which, under the influence of β-hCG, replace normal ovarian tissue. It is a purely solid mass, and its differentiation from solid ovarian neoplasms of stromal origin based on imaging is impossible [70]; however, these tumors are usually associated with androgen secretion, which may induce maternal and female fetus virilization, a clinical finding that may suggest the correct diagnosis [44]. It is important to identify these lesions since expectant management is recommended as they spontaneously regress during the early postpartum period [44]. The borderline ovarian tumor (BOT) is an epithelial tumor with a low rate of growth and low potential to invade or metastasize [64,65,66]. It is characterized by a specific proliferation pattern consisting of the stratification of the epithelial lining of the papillae, nuclear atypia, and mitotic activity, without stromal invasion [66]. Its incidence is 10–15% of all ovarian tumors [75,76]. The incidence of BOTs in pregnancy ranges from 0 to 8%. Imaging staging differentiates stage I (tumor limited to one or both ovaries), stage II (implants to pelvic peritoneum), and stage III (abdomino-pelvic peritoneal implants). Fertility-sparing surgery is frequently considered in these patients due to their young age, and laparoscopic surgery in BOTs is feasible in early-stage disease, but it is associated with a higher risk of recurrence if only cystectomy and then adnexectomy is performed [77].

**Figure 4:**
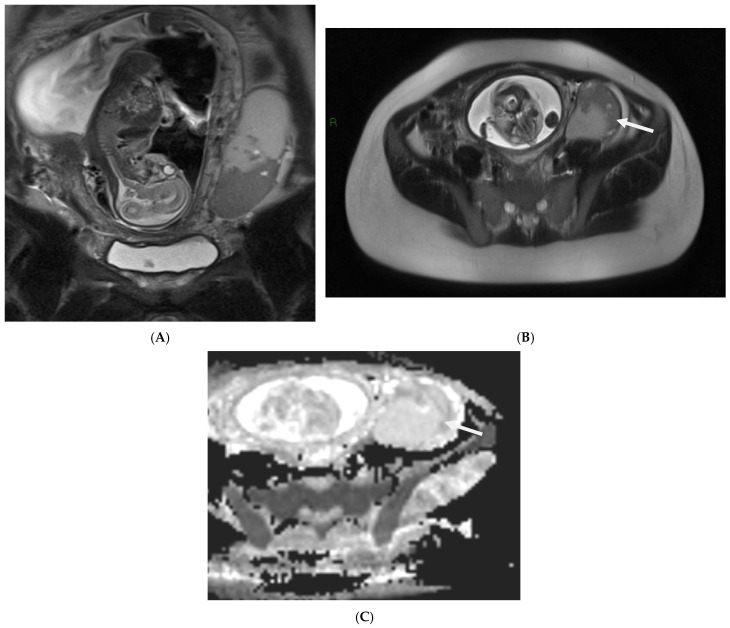
A 36-year-old woman at gestational week 32. Coronal (**A**) and axial (**B**) T2W Haste MR images show a large multilocular cystic mass in the left ovary. The signal intensity of the fluid varies, producing heterogeneous signal intensity, with suspected mural solid components (arrow). (**C**) Axial ADC map shows the mural nodule (arrow) with a relatively high ADC. A mucinous ovarian borderline tumor was found at surgery, performed after delivery.

It is reported that BOTs detected during pregnancy may be associated with more advanced disease and patterns of aggressiveness. In fact, 20% of pregnant patients in a recent study were diagnosed with FIGO stage II or III compared to a non-pregnant population where 90% of BOTs were diagnosed at FIGO stage I [78]. Mucinous histology BOTs were more frequent than serous BOTs, which were more common in the non-pregnant population (48 vs. 30%, respectively) (Figure 4).

Moreover, in the pregnant population, a considerable number of mucinous BOTs (21%) coexisted with more aggressive histology including intraepithelial carcinoma and microinvasion [78], and serous BOTs (41.2%) demonstrated micropapillary features, frequently bilateral with invasive implants. This histological aggressiveness could be explained by the presence of estrogen and progesterone receptors on BOTs, which are triggered during pregnancy because of higher concentrations of circulating hormones [39]. Close follow-up after the diagnosis of a suspected BOT during the third trimester of pregnancy is recommended by some researchers [78].

Decidualized endometrioma with intraluminal papillary vegetations demonstrating increased blood flow on Doppler ultrasound may mimic malignant ovarian tumors [65]. MRI may discriminate between benign endometrioma and ovarian malignant neoplasms. Typical decidualized endometrioma on MRI demonstrates hemorrhagic fluid and a variable amount of solid components, which demonstrate signal intensity similar to that of a normal placenta, and no restricted diffusion on high b values; in particular, computed DWI with b values of 1500 s/mm^2^ may be useful to distinguish decidualized endometriomas from ovarian malignancy [52]. The size of the solid components in the decidualized endometrioma is significantly lower compared with ovarian cancers, and they usually exhibit higher signal intensity compared to ovarian malignant lesions [64,65,66].

**Figure 5:**
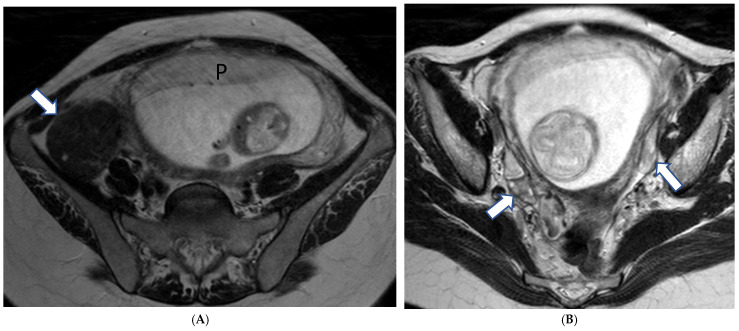
A 29-year-old pregnant woman at gestational week 21 with a suspicious right ovarian mass incidentally detected on routine second-trimester ultrasound: (**A**) Axial T2-weighted image shows a low signal intensity mass attached to the right side of the uterus typical of a pedunculated leiomyoma (arrow) (P: Placenta). (**B**) Axial T2-weighted image of the same patient at a lower level shows clearly the normal ovaries (arrows).

Another benign condition that can mimic malignancy at imaging is subserosal leiomyoma usually with red degeneration, a type of hemorrhagic infarction that often occurs during pregnancy due to venous thrombosis within the periphery of the mass or rupture of intra-tumoral arteries [67] (Figure 5).

Other features associated with an increased risk of ovarian malignancy in pregnancy include a tumor diameter of 10 cm or more (OR 11.2) and a tumor growth rate of more than 3.5 cm/week (OR 10.2) [68].

MR can be helpful for choosing the right timing for treatment since early surgery can induce luteal function loss and miscarriage, while late treatment may compromise oncologic outcomes [2,3,82]. Thus, a persistent ovarian mass in the second trimester or ovarian masses with MR features indicative of malignancy features, including septations and solid component nodules showing both low signal intensity on T2 and high b value DWI, should be surgically resected, outweighing potential benefits and risks and after careful patient consultation [80,81,83,84,85,86,87,88,89].

Surgery may also be indicated in large masses (diameter > 5 cm) to reduce the risk of ovarian torsion and/or poor obstetric outcomes [6].

### 3.3. Vulvar and Vaginal Tumors

#### 3.3.1. Epidemiology, Pathological Anatomy, and Clinical Management

Intraepithelial neoplasia-HPV related is the most frequent vulvar tumor in pregnancy [2].

When detected in pregnancy, vulvar cancer is most frequently seen in multiparous women, with the majority presenting in the second trimester.

Invasive cancer is rare, and nodal evaluation, in particular, inguinal nodal metastasis, is mandatory to assess disease severity and mortality. If mapping is negative for nodal disease, it is unnecessary to perform a lymphadenectomy, and its associated complications can be prevented.

Only case reports of vaginal cancer in pregnancy exist, with surgery during pregnancy offered as the treatment.

Deep aggressive angiomyxoma (DAM) arises in the deep soft tissue of the vulvovaginal region, perineum, and pelvis. It is a rare mesenchymal neoplasm that occurs mainly in women of reproductive age, with a median age between 30 and 40 years [90,91]. Since it derives from hormone-sensitive stromal tissues of the lower genital tract, the progression of this neoplasm is favored during pregnancy [90,91,92].

DAM can reach considerable dimensions during pregnancy, due to its hormonal dependence, given the presence of estrogen and/or progesterone receptors. Since the diagnosis is histological, clinical DAM is often misdiagnosed as other more frequent vulvar neoplasms, such as a vulvar cyst or lipoma. In most cases occurring in pregnancy, it is identified as a palpable vulvo-vaginal mass or swelling and the definitive diagnosis is usually made at histology.

In some patients, the tumor is identified as it increases in size during the postpartum period [92].

#### 3.3.2. Imaging

Transvaginal ultrasound is often used as the first diagnostic tool, identifying a solid hyper or -isoechoic mass with no defined margins, and showing increased vascularity on color Doppler [93,94].

The benefits of MRI imaging include superior contrast resolution and better soft tissue delineation of the primary vulvar tumor, and it is the most sensitive modality for detecting lymph node involvement.

Vulvar tumors appear as a solid mass, usually hypo-isointense on T1-WI, and moderately hyperintense on T2-WI in comparison to muscles. Malignant tumors show diffusion restriction on DWI.

A general recommendation is to perform a short-axis threshold of 10 mm to identify metastatic lymph nodes on MRI. The loss of fatty hilum and ovoid shape, rounded contour, irregular borders, and necrotic areas are highly suspected of nodal involvement from a tumor. Lymphadenopathies may also exhibit heterogeneous signal intensity on T2-weighted; DW imaging aids in nodal detection, since lymph nodes are easily identified by their high signal intensity.

**Figure 6:**
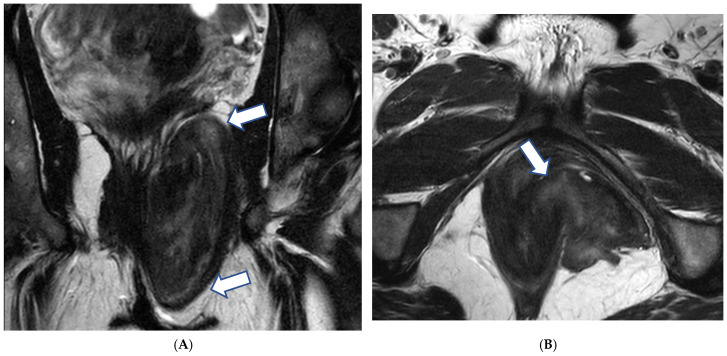
A 39-year-old woman, six months after a c-section presented with a left palpable vulvar mass. T2-weighted images in the coronal (**A**) and axial (**B**) plane demonstrate a large low T2 signal intensity mass occupying the left ischiorectal fossa (arrows in (**A**)) also invading the left side of the external sphincter (arrow in (**B**)). Biopsy showed the presence of a tumor of mesenchymal origin consistent with aggressive angiomyxoma.

MRI is the most appropriate imaging technique for evaluating the infiltration of the perineal and pelvic structures; typical appearances on magnetic resonance imaging include hypo-intensity on T1-weighted images hyper-intensity on T2-weighted images and non-restricted diffusion [93,94,95,96] (Figure 6).

### 3.4. Uterine Tumors

#### 3.4.1. Epidemiology, Pathological Anatomy, and Clinical Management

Uterine leiomyoma is the most common gynecologic tumor in a reproductive-age woman and is found in 10–20% of pregnancies [39,97]. Asymptomatic fibroids should be conservatively managed in pregnancy due to the risk of hemorrhage and fetal loss with surgery. Most fibroids do not enlarge significantly during pregnancy. The incidence of leiomyosarcoma in rapidly growing “fibroids” is only 0.27%; however, its preoperative diagnosis may be difficult.

A review of 15 uterine sarcomas in pregnancy reported that the most common symptoms at presentation were abnormal vaginal bleeding (40%), abdominal pain (33%), and an enlarged uterine mass (20%) [98,99]. The majority of patients underwent surgery during the third trimester or postpartum period (67%). The median survival for uterine sarcoma diagnosed in pregnancy has been estimated at 1.5 years.

The primary treatment of leiomyosarcoma is surgery and the standard procedure is total abdominal hysterectomy, and bilateral salpingo-oophorectomy. Myomectomy can be considered an alternative if the patient desires future pregnancies, but only if they understand and accept the risk of residual leiomyosarcoma and the risk of recurrence.

Endometrial cancer is very rare during pregnancy [50]. Moreover, because of its progesterone-dominant nature, pregnancy may have a protective effect on endometrial tumor growth. It is usually incidentally detected during curettage after miscarriage or delivery. Hence, the same treatment protocols as those used for non-pregnant patients are followed [50].

#### 3.4.2. Imaging

Pelvic ultrasound followed by magnetic resonance imaging (MRI) is the best imaging strategy for LMS. Sonographic features such as mixed echogenic and poor echogenic parts, central necrosis, and color Doppler findings of irregular vessel distribution in pelvic ultrasound can be suggestive of LMS; however, it may also be present in leiomyomas.

MRI findings of ill-defined margins, lack of calcifications, intra-lesional hemorrhage, intermediate-to-high areas mixed with dark areas on T2-weighted images, and high-restricted diffusion with low ADC values are features suggestive of malignancy [98].

Because the reported cases of endometrial cancer are so few, there is a lack of evidence regarding imaging diagnosis and work-up in these patients. When endometrial cancer is diagnosed, staging according to FIGO guidelines is recommended.

### 3.5. Placenta Tumors

#### 3.5.1. Epidemiology, Pathological Anatomy, and Clinical Management

Gestational trophoblastic disease (GTD) includes both benign and malignant gestational tumors, such as hydatidiform mole (complete and partial), invasive mole, choriocarcinoma, placental site trophoblastic tumor, and epithelioid trophoblastic tumor. The latter four entities are referred to as gestational trophoblastic neoplasia (GTN). These conditions are aggressive with a propensity to widely metastasize. GTN can result in significant morbidity and mortality if left untreated. Early diagnosis of GTD is crucial for prompt and successful management while preserving fertility.

Approximately 5% of cases of hydatidiform mole are followed by choriocarcinoma.

Only half the cases of choriocarcinoma arise from hydatidiform mole. An additional 25% of cases occur after normal pregnancies, and 25% arise after spontaneous abortion or ectopic pregnancy. At histological evaluation, choriocarcinomas have extensive necrosis and hemorrhage.

Solid placental masses are rare; chorioangiomas are the most common tumor of the placenta and are found in up to 1% of all placentas evaluated histologically. In up to 1:3500 births, chorioangiomas come to clinical attention [100].

These masses are usually >5 cm in size and may be associated with polyhydramnios, hydropic changes in the fetus, intrauterine growth restriction, and congestive heart failure of the fetus due to the vascularity of the mass. Chorioangiomas are benign tumors that can show intratumoral hemorrhage.

#### 3.5.2. Imaging

US characteristics of a complete hydatidiform mole include an enlarged uterus filled with a heterogeneous, mostly echogenic, mass with several hypoechoic foci (snowstorm appearance), and multiple small anechoic cystic spaces varying in size from 1 to 30 mm (cluster of grapes). Theca lutein cysts presenting as multiple large, bilateral, functional ovarian cysts can be present.

**Figure 7:**
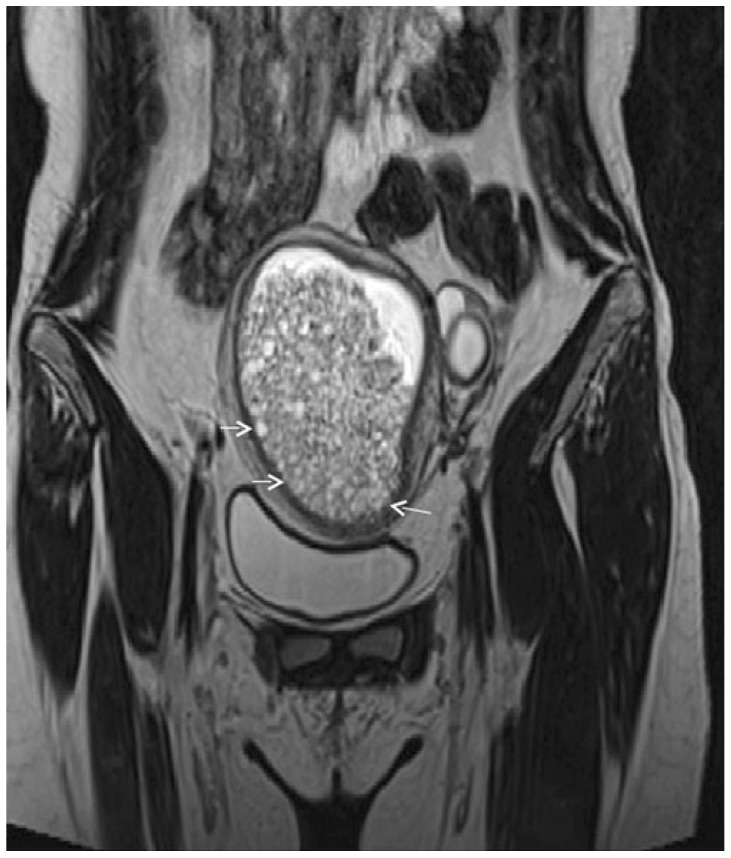
Coronal half-Fourier T2-weighted MR image in a patient with a hydatidiform mole. Coronal half-Fourier shows a large heterogeneous mass in the endometrial cavity; there is no evidence of myometrial invasion with the visualization of the normal dark myometrial line (arrows).

On T2-weighted images, a complete mole appears as a heterogeneous mass of high signal intensity that distends the endometrial cavity. Numerous cystic spaces may be present in the mass (Figure 7).

An invasive mole appears as a poorly defined mass with mixed signal intensity on T2-weighted images and a deep invasion of the myometrium; complete or partial disruption of the junctional zone may also be seen.

On T1-weighted images, the mass shows isointensity to the myometrium with scattered foci of high signal intensity because of the presence of hemorrhage. Molarlike structures appear as tiny cystic lesions within the well-enhanced zone of trophoblastic proliferation in a mass of the invasive mole. The use of color Doppler is helpful for differentiating a placental hematoma from a solid mass, such as a chorioangioma.

**Figure 8:**
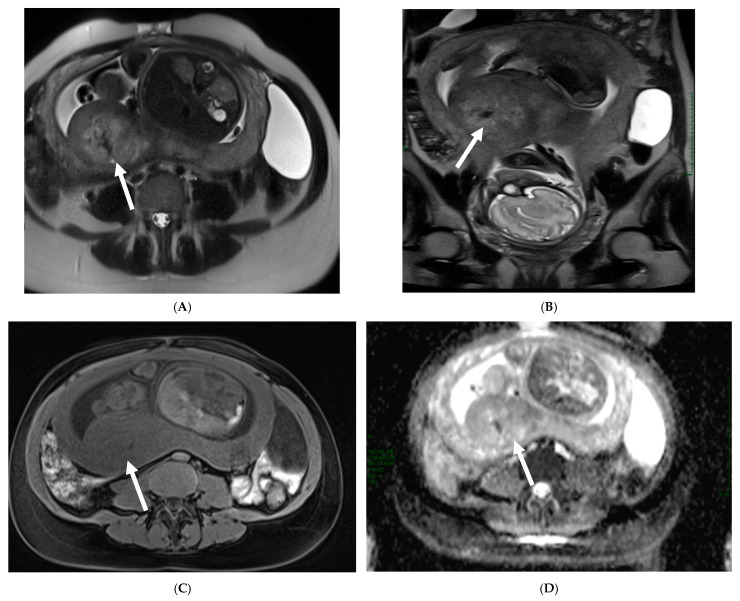
Placental chorioangioma in a patient at 26 weeks of gestational age and profuse vaginal bleeding. Coronal (**A**) and axial (**B**) T2-weighted HASTE and axial T1-weighted (**C**) images show an intrauterine rounded mass peripherally located and protruding from the fetal surface of the placenta (arrows). The mass is homogeneous and nearly isointense to the placenta on T1- and T2-weighted images, as well as on Diffusion Image (**D**) (arrows).

Chorioangiomas can be homogeneous and nearly isointense to the placenta on T1- and T2-weighted images. They are typically round in shape and may protrude from the placental surface [101] (Figure 8).

An increased T2 signal and increasing heterogeneity of the signal intensity have been reported in those masses with internal infarction or hemorrhage.

It is important to detect the presence of prominent vessels along the fetal surface of the mass given the potential for a hemodynamic impact on the fetus. If there are early signs concerning hydrops in the fetus, prompt notification of the ordering provider is recommended.

Although teratomas in the placenta are extremely rare, pregnancy outcomes are typically favorable.

Although fat and calcification can be readily identified at ultrasound, differentiation from an anomalous additional gestation or fetus acardius amorphous can be difficult, and MRI is useful if the diagnosis is uncertain. Acquisitions using fat saturation for bulk fat, and opposed-phase imaging to identify intravoxel fat, are helpful in the diagnosis of teratoma. The identification of fetal parts suggests an anomalous additional gestation is visible within the mass on T2-weighted and balanced steady-state free-precession imaging. Visualization of an umbilical cord (absent in teratoma) can also help in differentiation [101,102].

## 4. Conclusions

Gynecological cancer during pregnancy is a rare event and its management requires a careful multidisciplinary approach to balance the fetal and maternal risks.

Accurate imaging diagnosis is of critical importance for clinicians in order to optimize treatment planning. Imaging assessments strongly affect treatment options, quality of life, and future fertility preservation.

## Figures and Tables

**Table 1:** Samples of absorbed uterus dose by imaging modality.

Imaging Technique	Fetal Radiation Dose (mGy)
Chest X-ray	<0.01
Mammography (two planes, bilateral)	<0.01
CT of the head	<0.005 to 0.5
CT of the chest	0.001–0.66
CT of the abdomen/pelvis	8–25
18F-FDG PET/CT	10–50

Source: Reference [14]. Note, FDG = fluorodeoxyglucose.

**Table 2:** Magnetic resonance imaging protocol for evaluation of pregnant patients with gynecologic malignancies.

Sequence	Plane	Value
T2-W SSFSE (mandatory)	Axial (up to the level of renal ileum) Sagittal and Coronal	Overall view of pelvic and abdominal organs and minimizes fetal motion artifacts
High-resolution T2-W (mandatory)	Axial oblique plane (perpendicular to the cervical long axis), when cervical tumors are evaluated. True coronal plane (parallel to the uterine cavity), when adnexal masses are evaluated	Achieves optimal contrast difference between tumor and normal tissue
T1-W 3D fat suppression TSE or Dixon (mandatory)	Axial	Provides information on pelvic anatomy, hemorrhagic lesions, lymph nodes, and bone marrow. Fat suppression allows the diagnosis of hemorrhage vs. fat
DWI (0, 800, 1400 s/mm^2^) (mandatory)	Axial	Discriminates cancerous from normal tissue and detects lymph nodes
T2-W with fat suppression (optional)	Axial	Detects fluid collection and bone marrow changes
Steady-state sequences (optional)	Axial	Detects lymph nodes and vessel infiltration

Abbreviations: SSFSE—single-shot fast-spin echo; DWI—Diffusion-weighted imaging; 3D—three-dimensional; T1-W—T1-weighted; T2-W—T2-weighted; TSE—turbo-spin echo. Source: Reference [4].
